# Susceptibility of Human Lymphoid Tissue Cultured *ex vivo* to Xenotropic Murine Leukemia Virus-Related Virus (XMRV) Infection

**DOI:** 10.1371/journal.pone.0037415

**Published:** 2012-05-16

**Authors:** Marta Curriu, Jorge Carrillo, Marta Massanella, Elisabet Garcia, Francesc Cunyat, Ruth Peña, Peter Wienberg, Cristina Carrato, Joan Areal, Margarita Bofill, Bonaventura Clotet, Julià Blanco, Cecilia Cabrera

**Affiliations:** 1 IrsiCaixa-HIVACAT, Institut de Recerca en Ciències de la Salut Germans Trias i Pujol, Hospital Germans Trias, Universitat Autònoma de Barcelona, Carretera del Canyet S/N, Badalona, Barcelona, Spain; 2 Department of Otorhinolaryngology, Hospital Universitari Sant Joan de Déu, Passeig Sant Joan de Déu, Esplugues, Barcelona, Spain; 3 Department of Pathology, Hospital Universitari Germans Trias i Pujol, Carretera del Canyet S/N, Badalona, Barcelona, Spain; 4 Urology Department, Hospital Universitari Germans Trias i Pujol, Carretera del Canyet S/N, Badalona, Barcelona, Spain; 5 Institució Catalana de Recerca i Estudis Avançats, Barcelona, Spain; 6 Lluita contra la SIDA Foundation, Institut de Recerca en Ciències de la Salut Germans Trias i Pujol, Hospital Universitari Germans Trias i Pujol, Universitat Autònoma de Barcelona, Carretera del Canyet S/N, Badalona, Barcelona, Spain; Centers for Disease Control and Prevention, United States of America

## Abstract

**Background:**

Xenotropic murine leukemia virus-related virus (XMRV) was generated after a recombination event between two endogenous murine leukemia viruses during the production of a prostate cancer cell line. Although the associations of the XMRV infection with human diseases appear unlikely, the XMRV is a retrovirus of undefined pathogenic potential, able to replicate in human cells *in vitro*. Since recent studies using animal models for infection have yielded conflicting results, we set out an *ex vivo* model for XMRV infection of human tonsillar tissue to determine whether XMRV produced by 22Rv1 cells is able to replicate in human lymphoid organs. Tonsil blocks were infected and infection kinetics and its pathogenic effects were monitored

**Results:**

XMRV, though restricted by APOBEC, enters and integrates into the tissue cells. The infection did not result in changes of T or B-cells, immune activation, nor inflammatory chemokines. Infectious viruses could be recovered from supernatants of infected tonsils by reinfecting DERSE XMRV indicator cell line, although these supernatants could not establish a new infection in fresh tonsil culture, indicating that in our model, the viral replication is controlled by innate antiviral restriction factors.

**Conclusions:**

Overall, the replication-competent retrovirus XMRV, present in a high number of laboratories, is able to infect human lymphoid tissue and produce infectious viruses, even though they were unable to establish a new infection in fresh tonsillar tissue. Hereby, laboratories working with cell lines producing XMRV should have knowledge and understanding of the potential biological biohazardous risks of this virus.

## Introduction

Xenotropic murine leukemia virus-related virus (XMRV) was initially identified in some prostate cancer tissues [1] and while several studies confirmed the presence of the virus in human prostate cancer cells with similar [2]–[7], or lower prevalence [8]–[10], other authors have reported no evidence of the virus in patient samples [11]–[19]. Later, this retrovirus was also detected in blood samples of a high proportion of individuals with chronic fatigue syndrome (CFS) [20], in the respiratory tract of patients with or without a respiratory tract infection [21], and a similar polytropic murine leukemia viruses (MLV) was found even in a high proportion of CFS cases [22]. Nevertheless, several subsequent studies failed to identify XMRV in CFS patients or healthy donors [23]–[30], and even blood samples from CFS patients previously reported to contain XMRV sequences were retested and were identified as XMRV negative [27], [31], [32]. These data have resulted in the retraction of Lombardi et al. and Lo et al. papers [33]–[35].

In order to explain the positive results, several studies suggested that the detection of XMRV sequences in human samples was a result of contamination of laboratory reagents with mouse DNA [36]–[44], with DNA from the chronically infected DU145 cell line [45], [46] or with a XMRV plasmid DNA [33]. Finally, the assertion that XMRV is circulating in human population has been challenged by the report showing that XMRV was generated by a recombination event between two endogenous MLVs during *in vivo* tumor passaging in mice [47], which yielded the popular prostate cancer cell line 22Rv1 [48].

**Figure 1:**
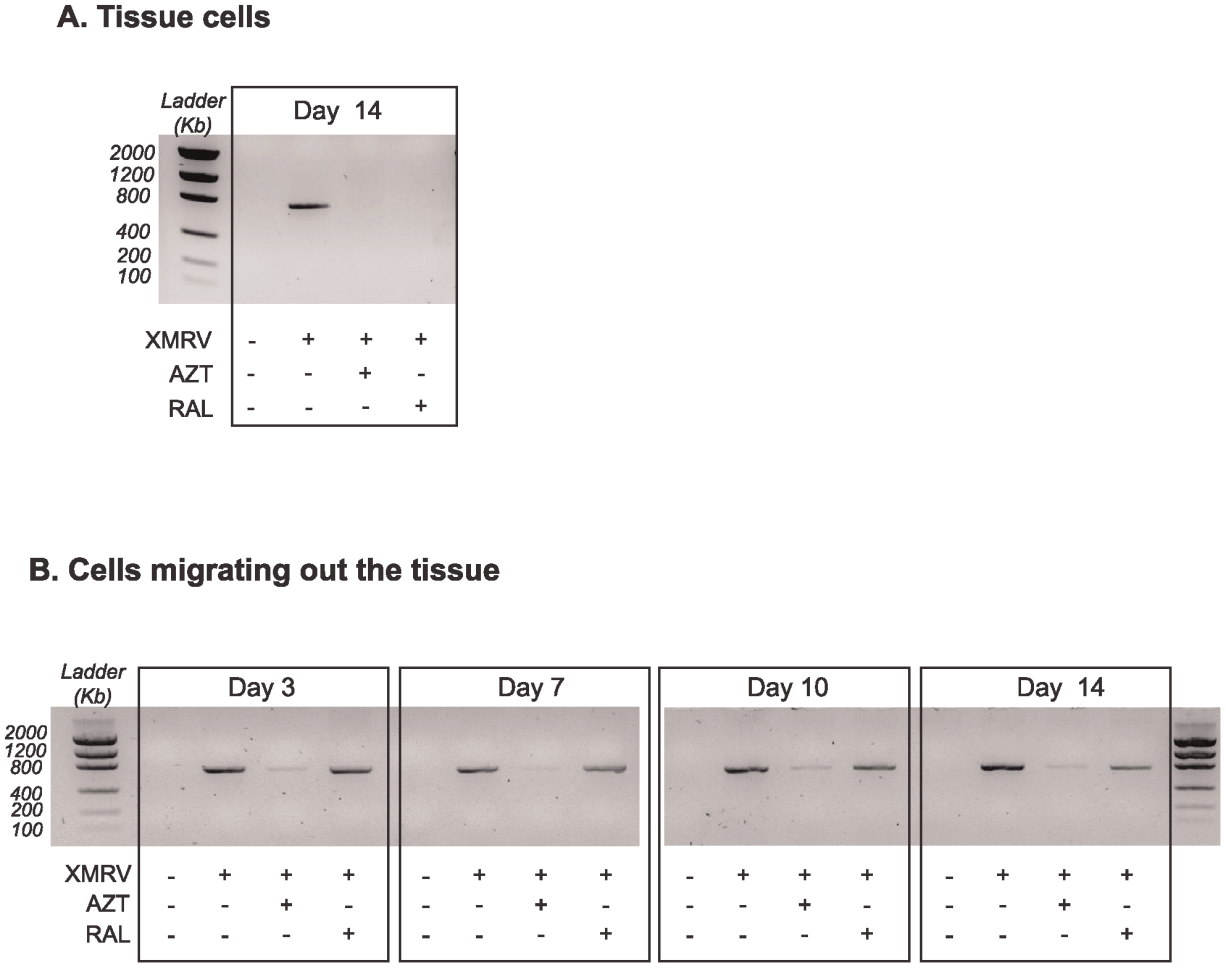
Detection of proviral XMRV DNA in human lymphoid tissue. DNA was amplified by a single-round conventional PCR using a XMRV *gag* specific primer set and the PCR fragments were analysed on a 2% agarose-gel. A) XMRV-*gag* fragments in isolated tissue cells at the last day of the experiment in presence or absence of the antiretroviral inhibitors AZT and RAL. B) Proviral DNA content in cells migrating out of the tissue at different time points over the culture in absence or presence of AZT or RAL. PCR results from a representative experiment are shown.

**Figure 2:**
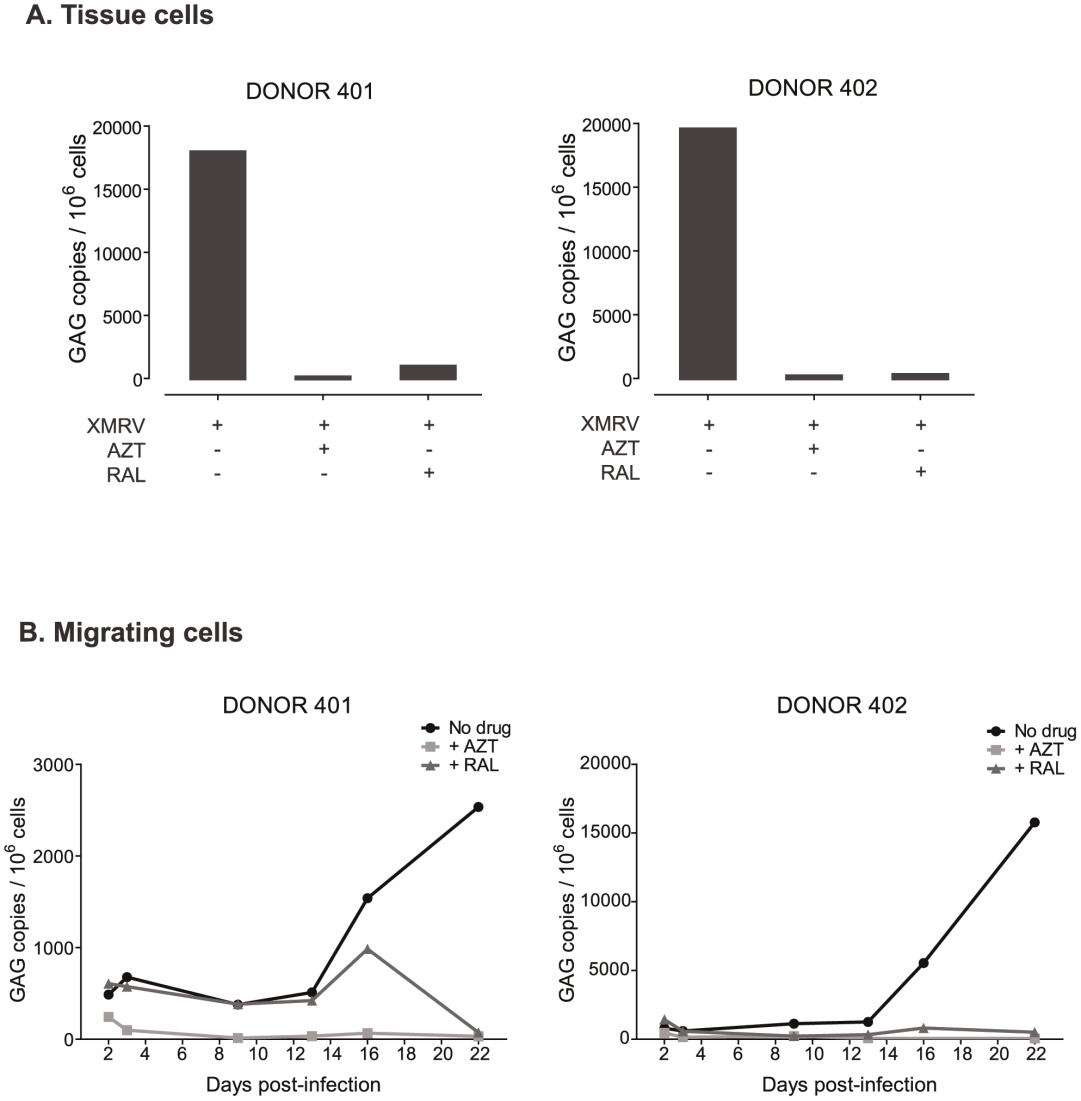
Quantitative time course of XMRV *gag* sequences of *ex vivo* infected tonsillar tissue. Isolated DNA was amplified by quantitative real-time PCR and the absolute XMRV copy numbers were obtained by normalizing the results with the values obtained with the single copy CCR5 gene in each sample. Each sample was run in duplicate. A) Absolute XMRV DNA content in tissue cells, after 22 days of infection, in the presence or absence of two antiretroviral drugs, AZT and RAL. B) XMRV DNA content in cells migrating out of the tissue collected from days 2 to 22 post-infection in the presence or absence of the indicated inhibitors.

**Figure 3:**
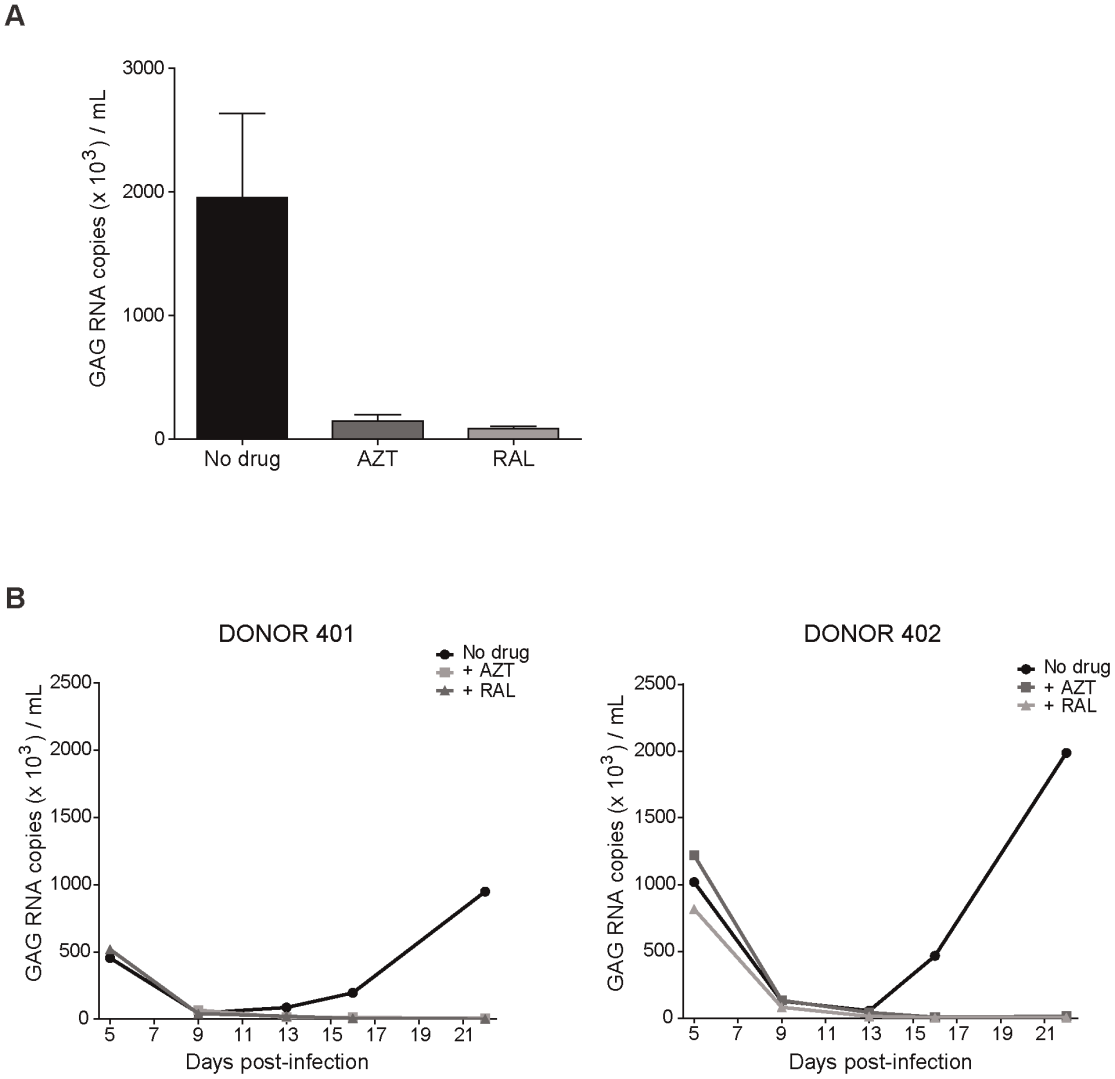
XMRV RNA released by *ex vivo* human lymphoid tissue infected with 22Rv1 supernatant. Blocks of tonsillar tissues were infected *ex vivo* with XMRV from 22Rv1 cells supernatant. Culture medium bathing the tissue blocks was changed every 3–4 days and analysed for the presence of XMRV RNA using a quantitative real-time RT-PCR. A) Cumulative amount of XMRV RNA released between days 9 and 22 post-inoculation in infected cultures in the presence or absence of the antiviral drugs AZT and RAL. The graph represents the viral RNA content in two donors (mean ± SEM). B) Kinetics of XMRV RNA released by infected tonsillar tissue and the effect of the addition of antiviral drugs to the culture.

**Figure 4:**
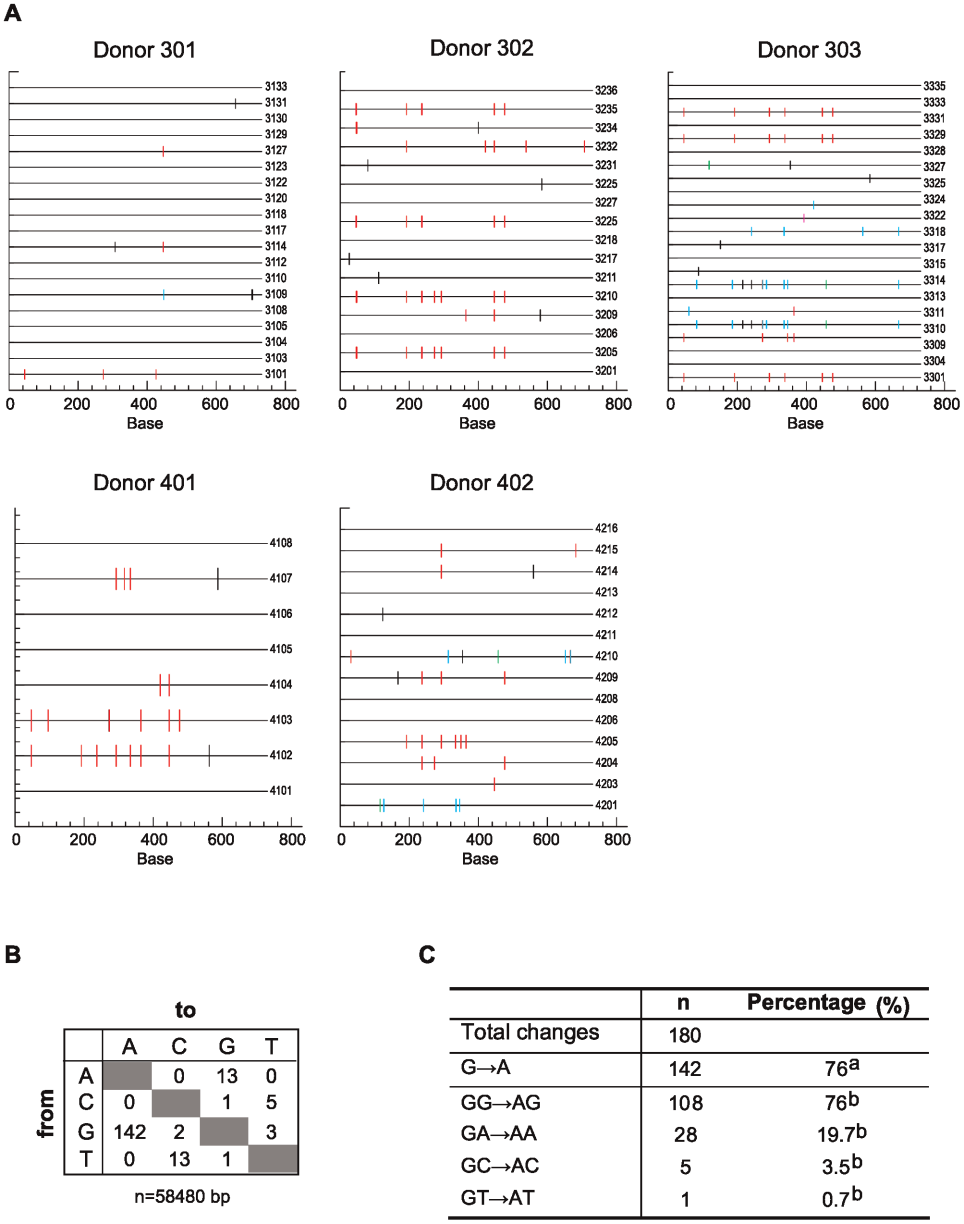
Hypermutation of XMRV in infected human lymphoid tissue. DNA from tonsillar tissue infected with XMRV was amplified, cloned and sequenced. A) Graphic representation of the changes (compared to XMRV VP42) presents in the XMRV *gag* region. The mutations presents in clones from 5 different donors are shown. Each mutation is denoted by a vertical line marked onto a single horizontal line representing the entire amplicon, color coded with respect to dinucleotide context: GG→AG (red). GA→AA (cyan), GC→AC (green), GT→AT (magenta), and non G→A (black). Analysis was performed using the HYPERMUT program [91]. B) Preferences of nucleotide substitutions from all the obtained sequences, “n” denotes the total number of sequenced base pairs. C) Summary of the observed changes at the different dinucleotide context. “n” denotes the number of mutations;^ a^Percentage of the total number of changes; ^b^Percentage of the number of G-to-A changes.

**Figure 5:**
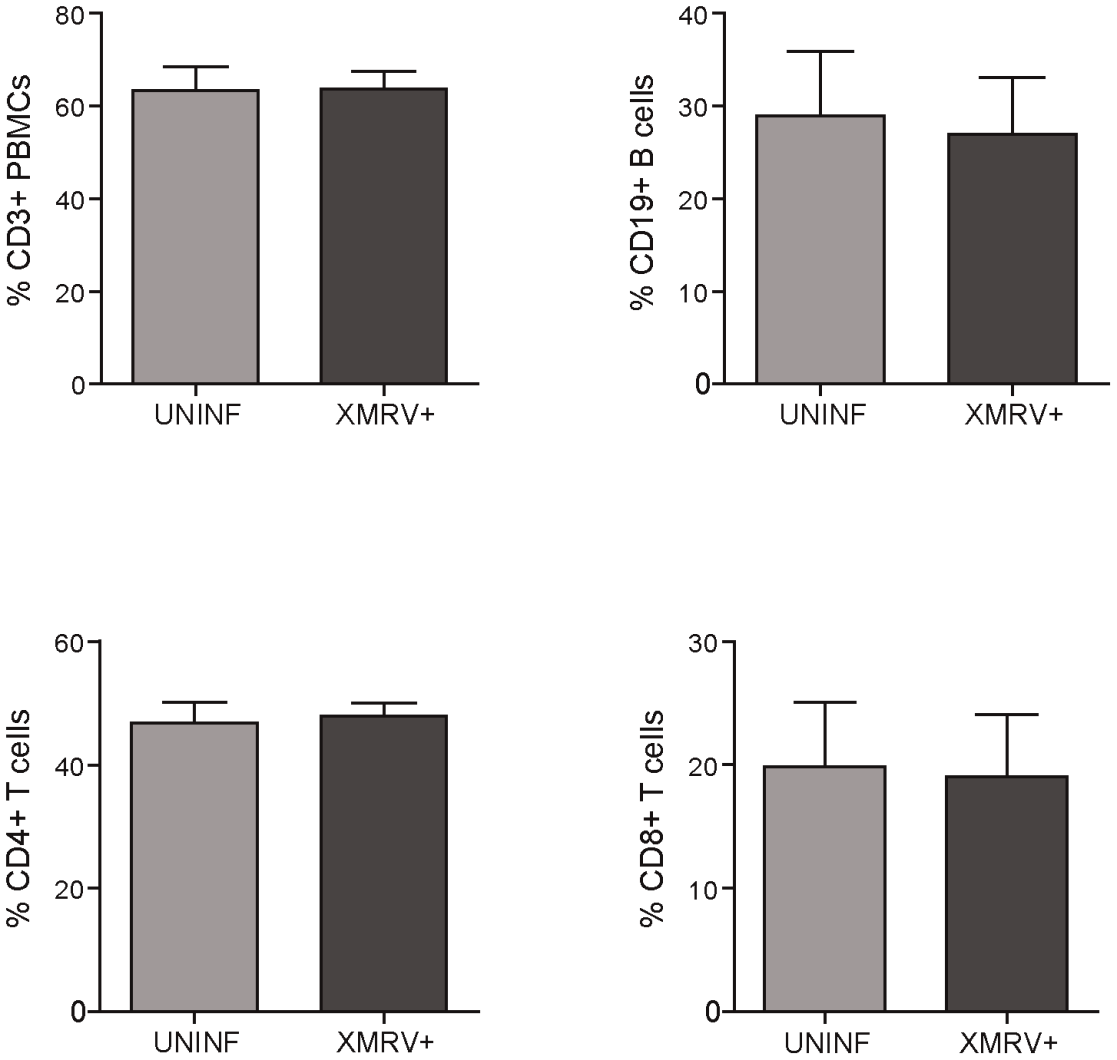
Lymphocyte populations in uninfected and XMRV infected lymphoid tissue. Tissue cells at day 9 post-infection were isolated, stained with monoclonal antibodies and analyzed using flow cytometry. Percentages of T (CD4^+^ and CD8^+^) and B cells in uninfected (XMRV^−^) and XMRV infected (XMRV^+^) tissues are shown. Data are means ± SEM from 5 independent infected tonsils.

**Figure 6:**
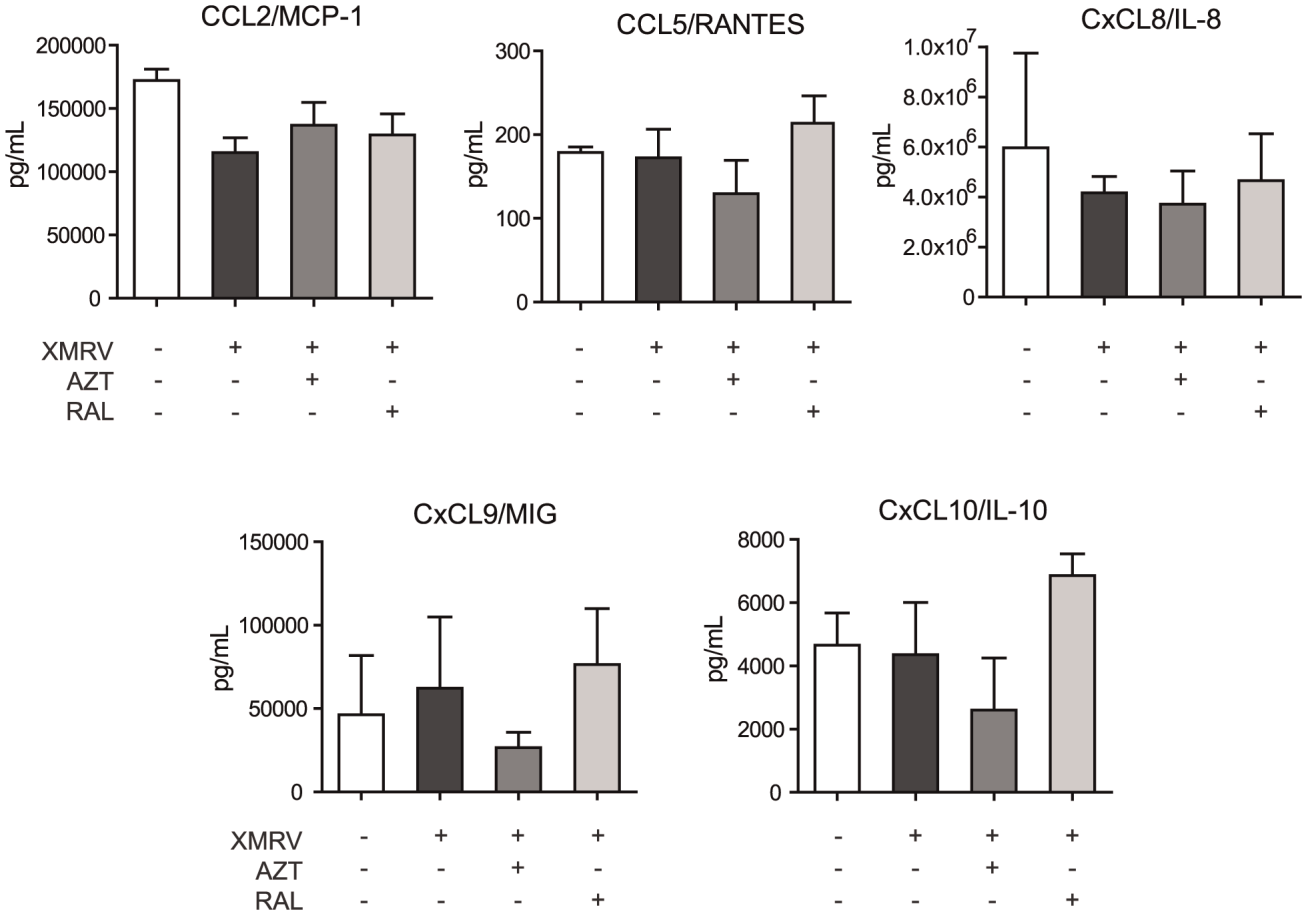
Chemokine production of tonsillar tissues infected *ex vivo* with XMRV. Tonsillar tissue was infected with XMRV and chemokine concentrations were measured using Cytometric Bead Array (CBA) in samples of culture medium collected at days 2, 5, 9, 13, 16 and 22. Data are presented as cumulative amount of released chemokines over the 22 days of culture in the absence or presence of AZT and RAL. Two separate experiments using two donors were carried out. Data presented are means ±SEM.

**Figure 7:**
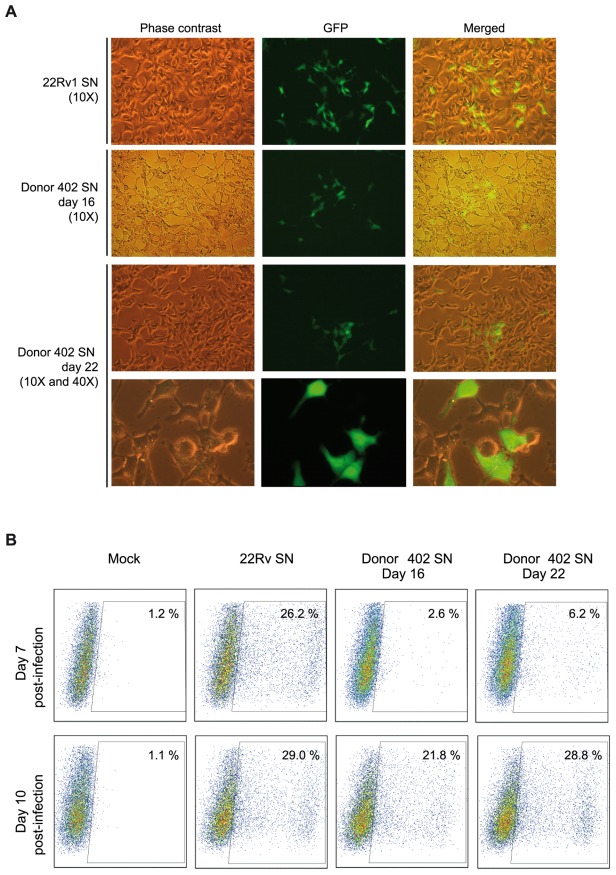
GFP-expression in DERSE XMRV indicator cells infected with tonsil supernatants. DERSE XMRV indicator cells were infected with tonsil supernatants harvested at day 16 and 22 post-infection (donor 402, see Figure 3B) and with a XMRV stock (22Rv1 supernatant) as positive control. A) DERSE-GFP positive cells were visualized by conventional phase contrast microscopy and fluorescence microscopy. Phase contrast image (left panel), GFP fluorescence image (central panel) and GFP/phase contrast merge image (right panel). Data are representative of two independent experiments. B) Quantitative assessment of DERSE GFP-positive cells. After 7 and 10 days post-infection (D7 p.i. and D10 p.i.) DERSE infected cells were harvested and the percentage of GFP-expressing cells was analyzed by FACS analysis.

**Figure 8:**
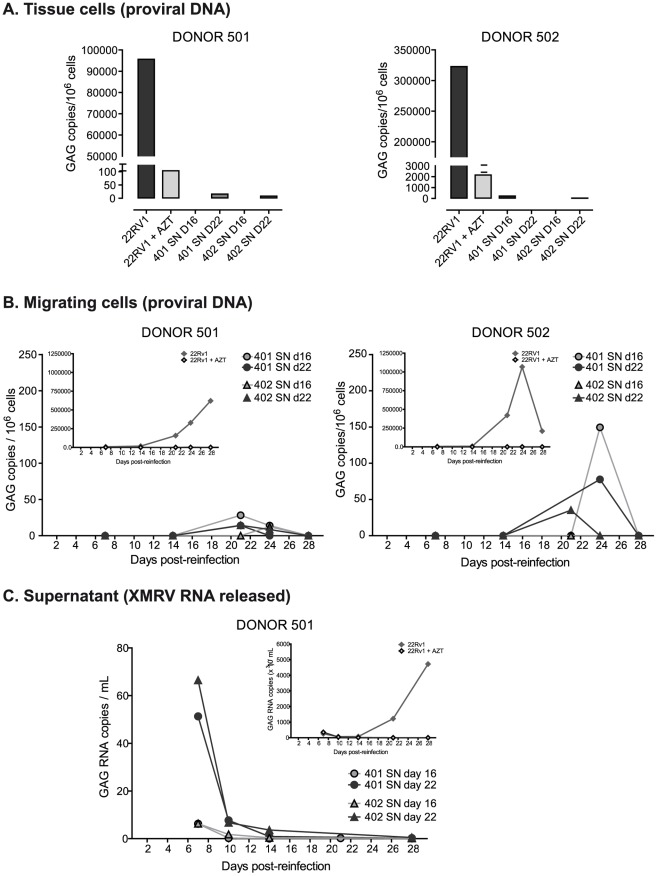
Re-infection of human lymphoid tissue with tonsil supernatants. New histocultures from two different donors were prepared and infected at day 0 and 1 with a XMRV stock (22Rv1 supernatant) and supernatants harvested at day 16 and 22 post-infection (donor 402, Figure 3B). The positive control (22Rv1 supernatant) was cultured in the presence or absence of AZT. A) Proviral XMRV DNA analyzed by quantitative real-time PCR in tissue cells isolated after 28 days post-reinfection. Each sample was run in duplicate. B) Absolute proviral XMRV DNA content in cells migrating out of the tissue collected from days 7 to 28 post-reinfection. C) Kinetics of XMRV RNA released by lymphoid tissue into the medium analyzed by real-time qRT-PCR.

The 22Rv1 (ATCC CRL-2505) cell line was established from human prostate tumor explants after serially passing through athymic nude mice around 1991 [48]–[50] and has been widely used in laboratory experiments in prostate cancer research with over 198 citations in PubMed. Electron microscopic analysis of culture medium from this cell line revealed the presence of gammaretrovirus-like particles, further identified as a xenotropic murine leukemia virus-related virus (XMRV) showing multiple integrated copies and a high-level of viral production [51].

Despite the discovery of the origin of the virus and the accumulation of data showing that the virus is not present in the general human population, XMRV is a novel replication-competent virus and could be a potential human pathogen since it has been shown to successfully infect many human cell types *in vitro* [6], [52], [53] including PBMCs and neuronal cells [17], [54], [55]. Indeed, contamination of vertebrate cell lines with retroviruses has been widely described, being gammaretroviruses the most frequent contaminants [56]–[62]. About 60 copies of MLV sequences are present as endogenous proviruses in mouse genomes [63] from which up to 15 copies are related to infectious xenotropic murine leukemia viruses (XMLV) [64]. The XMLV are type C retroviruses from mouse which can infect human or other foreign species [65]–[67] and have been identified in human cultures derived after xenografting immune compromised mice [68]–[71]. Recently, the presence of several MLV strains in over one fourth of xenograft cell lines has been documented [72], some of them releasing large number of infectious virions. This is the case of 22Rv1 cells, a high-titer XMRV producing cell line which should be carefully considered from the standpoint of possible horizontal spread to other human cells and biohazard of unknown potential to laboratory personnel [51]. Regarding the virus transmission to other cell lines, two cell lines that have become infected with XMRV in the laboratory by a contamination with the 22Rv1cell line have already been reported, confirming the highly infectious nature of this virus [72], [73]. Concerning the potential biohazards to laboratory personnel involved in cell culture facilities, it is important to explore XMRV infectivity and replication capacity in humans.

The infectivity of XMRV in humans may be strongly limited by innate host restriction factors such the APOBEC (apolipoprotein B mRNA-editing catalytic polypeptide) family, which play an important role in viral tropism, establishment of viral infections *in vivo* and successful spread of viruses. The family of APOBEC proteins, including APOBEC 3G and APOBEC 3F, have been implicated in the inhibition of a variety of retroviruses (HIV, SIV, HTLV and MLV) [74]–[78] and retrotransposons [79], [80]. These proteins are cytidine deaminases, which when incorporated in viral particles alter the nascent retroviral DNA leading to massive G-to-A hypermutation of the viral genome [81], [82]. In the case of XMRV, it has been shown that its replication is highly sensitive to APOBEC proteins and tetherin [52], [54], [83]–[85]. XMRV was hypermutated in A3G/A3F-expressing cell lines and in cultured human PBMCs, where replication was potently inhibited [54], [83]. However, despite these observed *in vitro* restrictions, infectious XMRV was recovered from infected PBMCs when cocultured with a permissive cell line, suggesting that even if PBMCs do not support a productive infection, they may potentially act as a source of infectious XMRV *in vivo* [17], [54].

In the absence of any known human infection, information about the infectivity and replication capacity in humans is needed. Until now, animal models have been used for the study of the natural history of XMRV infection, although the infection of two non-human primate species has yielded conflicting results [86], [87]. Organ cultures provide a versatile experimental system for studying the primary events surrounding virus transmission and the initiation of viral infections in human cells. It has been shown that human lymphoid tissue cultured *ex vivo* is a suitable model for HIV and HHV-6 pathogenesis [88]–[90]. In this system, HIV-1 infection results in cell activation and massive CD4^+^ T-cell depletion

The present study focuses on the characterization of infection of XMRV derived from the 22Rv1 cell line in lymphoid tissue *ex* vivo. We found that XMRV could infect human lymphoid tissue and infectious viruses were recovered capable to infect a DERSE XMRV indicator cell line, despite viral genomes were highly edited by APOBEC. However, the amount of virus released into the culture supernatant was not sufficient to establish a new infection in lymphoid tissue. The infection appears to have no pathogenic effects in lymphocytic populations or in the level of inflammatory chemokines, nonetheless, laboratories working with cell lines producing XMRV should have knowledge and understanding of the potential biological biohazardous risks of this virus.

## Materials and Methods

### Cells, viruses and antiviral agents

22Rv1 cells were obtained from the American Type Culture Collection (ATCC) and Detectors of Exogenous Retroviral Sequence Elements (DERSE) XMRV indicator cell line was kindly provided by Dr. Vineet N. KewalRamani (National Cancer Institute, Frederick, Maryland, USA). Both cell lines were maintained in RPMI 1640 medium with 10% FBS (Invitrogen, Spain). Cell-free XMRV was harvested from 22Rv1 supernatant, clarified by centrifugation; aliquots were stored at −80°C until used. Zidovudine (AZT) was purchased from Sigma-Aldrich (Spain) and Raltegravir (RAL) was obtained through the AIDS Research and Reference Reagent Program, Division of AIDS, NIAID, NIH; from Merck & Company, Inc.

### XMRV infection of human lymphoid tissue *ex vivo*

Human tonsils were obtained from patients undergoing therapeutic tonsillectomies. All procedures followed the Helsinki Declaration in 1975, as revised in 1983, and were approved by the Ethics committee of the Hospital Germans Trias i Pujol. All individuals provided their written informed consent. The specimens were dissected into ∼2–3 mm^3^ blocks, and cultured in RPMI 1640 medium supplemented with 15% fetal bovine serum, 1 mM sodium pyruvate (Invitrogen Life Technologies, Spain), 0.1 mM minimal essential medium with nonessential amino acids (Invitrogen) and a mixture of antibiotics on top of collagen sponge gels at the medium-air interface as previously described [88].. Nine individual blocks were placed on the gelfoam in a well of a six-well plate. Lymphoid tissue blocks were left uninfected or were infected with 5 µL of XMRV stock (4×10^8^ RNA copies/mL) for each tissue block. Cultures were conducted in the absence or the presence of reverse transcriptase or integrase inhibitors, AZT (10 µg/mL) or RAL (5 µg/mL), respectively. Fresh drug was added at every medium change. Each experimental condition was composed of three wells for a total of 27 blocks whose culture media were pooled. Culture medium was harvested every 3–4 days, centrifuged and both, the pellets with the cells migrating out of the tissue, and the clarified supernatant were frozen at −80C until use. On days 14 to 22 after infection, cells were mechanically isolated from uninfected and infected tissue blocks and immunophenotyped. Cell Pellets were also stored at −80°C for further analyses.

### XMRV DNA detection

Viral infection was evaluated at different time points in the cells migrating out of the tissue and in tissue cells at the end of the culture by analyzing the presence of viral DNA by conventional PCR. DNA was isolated using QIAamp DNA Blood kit (Qiagen, Spain) according to the manufacturer's instructions, and *gag* gene was amplified as previously described [20]. Briefly, PCR was conducted with 5 µL of extracted DNA and the primers 419F and 1154R using Accuprime Taq DNA Polymerase (Invitrogen, Spain). The cycles were 10 min at 94°C (30 sec at 94°C, 30 sec at 57°C, 1 min 68°C)×40 cycles and 10 min at 68°C. Then, samples were electrophoresed in 2% agarose gels containing SYBR safe DNA in TAE buffer.

### Quantitative Real-Time PCR

To quantify the absolute XMRV DNA copy numbers, standard plasmids for XMRV-*gag* and for the single-copy CCR5 gene were used. Both plasmids were constructed by conventional PCR using AmpliTaq High Fidelity (Invitrogen, Spain) and the following primers: Q445F and Q528R previously described for the detection of XMRV *gag* [6] and CCR5-576F (5′-TCATTACACCTGCAGCTCTCATTT-3′) and CCR5-726R (5′- ACACCGAAGCAGAGTTTTTAGGAT-3′) for CCR5 detection. The two PCR products purified with QIAquick PCR Purification Kit (Qiagen, Spain) were cloned into a PCR-Script Amp Cloning Kit (Agilent, Spain) according to the manufacturer's instructions. The plasmids were purified by standard methods, their concentrations were measured using a fluorometer and the corresponding copy numbers were calculated. Quantitative real-time PCR was performed with DNA isolated from the cells migrating out the tissue at different time-point during the culture and in tissue cells at the end of the culture in duplicate, using a TaqMan Universal Master Mix (Applied Biosystems, Spain), the primers described above and the probes: F480PRO; (5′-FAM-ACAGAGACACTTCCCGCCCCCG-MGBNFQ-3′) and CCR5-661T (5′-VIC-CTGGTCCTGCCGCTGCTTGTCA-TAMRA-3′) to detect XMRV *gag* and CCR5, respectively. A ten-fold serial dilution of the two standard plasmids, ranging from 1×10^10^ to 1×10^−1^ copies/reaction was used in duplicate to construct standard curves. All real-time PCR reactions were performed on a ABI Prism 7000 (Applied Biosystems, Spain).

### Quantification of viral RNA released

Quantitative measurements of XMRV viral RNA in culture supernatants were done using a quantitative real-time RT-PCR assay. Total RNA was extracted from 280 µL of culture supernatant at different time points during the culture (QIAamp Viral RNA kit, Qiagen, Spain) and one-step quantitative reverse transcription PCR was performed using the Ag-path-ID One-Step RT-PCR Kit from Ambion (Applied Biosystems, Spain) using the primers, probes and standard curves described above.

### G-to-A hypermutation of XMRV proviruses in infected tissue cells

PCR products obtained by conventional PCR from tissue cells (see above) were cloned using the PCR-Script Amp Cloning Kit. Over 10–30 colonies were selected for each product and sequenced using the Big-Dye Terminator Cycle Sequencing and the ABI 3100 sequence analyzer (Applied Biosystems, Spain). All sequences were assembled, aligned and edited using the Sequencher v.4.2 and GeneDoc v.2.6.001 software. The alignments were uploaded to the Hypermut tool on the Los Alamos National Laboratories website (http://www.hiv.lanl.gov/content/sequence/HYPERMUT/hypermut.html) to identify hypermutated sequences [91].

### Infectivity of newly produced viruses by infected tonsils

To analyze infectivity of supernatants from XMRV-infected tonsils we used two different approaches. First, infection of the DERSE XMRV indicator cell line was performed. The development of DERSE cells involved the transfection of LNCaPs cells with a MLV reporter vector encoding puromycin resistance and a CMV enhancer/promoter driven GFP reporter gene whose transcription was antisense to the vector mRNA (KewalRamani VN, unpublished data). DERSE XMRV indicator cells were plated at a density of 40,000 cells/well in 48-well plates and allowed to grow overnight. Next day, the cells were infected with culture supernatant (50 µL) of previous tonsils infected and harvested at days 16 and 22 post-infection (donor 402). As a positive control we infected one well with the 22Rv1 supernatant stock. The culture was splitted at days 3, and 7 and maintained for 10 days. The percentage of GFP-expressing cells was evaluated by fluorescence microscopy and flow cytometry. In addition, new tonsils histocultures were re-infected with supernatant harvested at day 16 and 22 (donor 401 and 402) from previous histocultures infections. The infection was done at day 0 as described above although here the tissue was re-infected with the same amount of virus at day 1 of culture. The culture was maintained 28 days and culture medium, cells migrating out of the tissue and tissue cells were harvested as previously described.

### Immunophenotypic characterization of tissue lymphocytes

Single cell suspensions were prepared as described above. Tissue lymphocytes at the end of the culture were immunophenotyped using monoclonal antibodies against the following surface antigens: CD3, CD4, CD8, CD45RO, CD38 and HLA-DR for the analysis of T cell, and CD19, IgD, CD38, CD27, from Becton Dickinson, Barcelona, Spain) and CD10 for B cells (from eBioscience, Barcelona, Spain). Multicolor analysis was performed with a LSRII flow cytometer (Becton Dickinson). Data were acquired with the BD FACSDiva software and analyzed with Flow Jo software.

### Flow cytometric analysis of chemokines

Culture supernatants clarified by centrifugation and collected at different time points during culture were assayed using a Cytometric Bead Array kit (CBA, BD Biosciences, Spain). Chemokines (CXCL8/IL-8, RANTES, CXCL9/MIG, CCL2/MCP-1, and CXCL10/IP-10) were measured using the Human Chemokine kit according to the manufacturer's instructions using a LSRII flow cytometer (Becton Dickinson).

## Results

### XMRV infection of human lymphoid tissue

To evaluate the ability of XMRV to infect human lymphoid tissue, infectious XMRV harvested from prostate carcinoma cell line 22Rv1 was used to infect tonsillar explants *ex vivo*. After 14 days post-infection, we analyzed the presence of XMRV *gag* proviral DNA in tissue blocks. Cells were mechanically isolated from uninfected and infected tissue and the presence of XMRV *gag* proviral DNA was clearly observed in infected tissue cells by conventional PCR at the end of each experiment (Figure 1A). The specificity of the infection was evaluated using AZT and RAL, two antiretroviral drugs known to inhibit XMRV reverse transcriptase (RT) and integrase, respectively [92], [93]. Both drugs completely inhibited XMRV DNA detection in tissue cells, indicating that infection is the result of specific reverse transcription and integration processes (Figure 1A). To assess the kinetics of the infection we quantified the XMRV proviral DNA in cells migrating out the tissue, which were collected every 3–4 days. After 3 days of culture, migrating cells were already XMRV positive and remained positive until the last day tested (Figure 1B). The RT inhibitor AZT markedly inhibited the presence of XMRV proviral DNA in all time points. Conversely, modest effects were observed after 7 or 14 days in the presence of the integrase inhibitor RAL (Figure 1B). Cells from the mock-infected tissue were negative in all cases.

### Increase in proviral XMRV DNA content during the culture of human lymphoid tissue

Once we confirmed the entry and the reverse transcription of XMRV in the human lymphoid tissue, we accurately evaluated the level of the infection by setting up a quantitative real-time PCR to measure the absolute XMRV DNA content. Total XMRV *gag* copies per 1×10^6^ cells were obtained for several tissues by CCR5 normalization after 22 days of infection (Figure 2). A clear increase in the amount of proviral DNA present in tissue cells was observed when comparing with the histocultures infected in the presence of antiretroviral drugs. The treatment with AZT and RAL resulted in almost complete inhibition (>97%) at most time points and donors tested (Figure 2A). In migrating cells, the increase in the infection was detected after 9–10 days of culture, although the proviral DNA content underwent a significant increase at later points (after 16 days post-infection, Figure 2B). The presence of AZT in the culture reduced proviral DNA level to negative values from day 3 to the end of cultures. However, in the presence of RAL, proviral DNA values were comparable to the infected tissue without drug and remained stable until day 7–13, declining at the last days of culture (Figure 2B). This different behaviour in the inhibition between both drugs could be explained by the fact that our PCR system detected not only the integrated DNA, but also unintegrated DNA. AZT would totally block viral DNA synthesis, resulting in decrease of both total and integrated DNA. On the contrary, when an integration inhibitor like RAL is used, unintegrated viral DNA accumulates in the nuclei in the form of 1-LTR and 2-LTR circles [94]. These episomal forms may explain the detection of XMRV DNA at an almost constant value over time in our infected tissue in the presence of RAL.

### XMRV RNA detection in the supernatant of infected histocultures

The observed increase in viral DNA content in both, tissue and migrating cells, indicated that XMRV might establish a productive infection in our histoculture model. To evaluate this possibility, XMRV RNA released into the medium, starting on day 5 post-infection, was quantified in culture supernatants by using real-time qRT-PCR. The cumulative production in the infected cultures from day 9 to day 22 post-infection is shown in Figure 3A. XMRV RNA was mainly detected in infected tissues that were cultured without antiviral drugs. The total production of viral RNA in AZT and RAL containing cultures constituted only the 8 and 4%, respectively of the infected tissues without drug (Figure 3A). The evaluation of replication kinetics revealed a decrease in RNA viral levels up to day 9 in cultures with or without drug, representing the washing out of the starting viral inoculums (Figure 3B). Thereafter, RNA virus release experienced a continuous increase over time in infected tissue without drug from day 9 to the last day of culture, reaching levels up to 2×10^6^ copies/mL after 22 days of culture. This increase in RNA virus release, ranging from 16- to 21-fold in the different tested donors, was completely blocked by AZT or RAL (Figure 3B). These findings could indicate that XMRV production, which is sensitive to RT and integrase inhibitors, occurs in tonsillar tissue.

### Host restriction of XMRV in human lymphoid tissue

A severe restriction in the spreading of the XMRV infection has been shown in PHA-activated human PBMCs [54]. Since human lymphocytes express restriction factors, such as APOBEC 3G and 3F, [84], [95] we sought to determine whether these factors impacted on XMRV replication in tonsils. DNA from infected tissue cells at the last day of the experiment was isolated and a 731-base pair fragment of the XMRV *gag* region was amplified. The PCR products were cloned and their DNA sequences were compared to the XMRV VP42 consensus. A total of 80 clones derived from 5 different donors were analyzed (Figure 4A). An initial analysis revealed that 46 sequences (57.5%) had mutations, although only 32 (40%) of them harbored more than one mutation. To identify the hypermutated sequences we used the LANL Hypermut 2.0 program [91], using a p-value<0.05. Compared to XMRV VP42, a total of 15 (18.8%) sequences were identified as hypermutated, with a range of 0 to 31.2% among different donors. In total, 180 changes were detected with a mutation frequency of 3×10^−3^/nt (180 mutations for 58480 nucleotides sequenced) of which 142 were G-to-A changes (Figure 4B). Substitutions in the GG dinucleotide context (GG-to-AG), which is the specific target site for APOBEC 3G [81], [82], were more prevalent (76%) than in the APOBEC 3F-preferred context (GA-to-AA) (20%), suggesting that XMRV in our *ex vivo* infection is mainly restricted by APOBEC 3G (Figure 4). At the amino acid level, mutations caused both, changes in the amino acid sequence and introduction of stop codons.

### Pathogenic effects of XMRV infection

To further characterize the XMRV infection in tonsillar tissue, we investigated possible pathogenic effects. It has been shown that HIV-1 severely depletes CD4^+^ T-cells in *ex vivo* infected human lymphoid tissue [88], [89], with a depletion of 77 and 97% of CD4^+^ T-cells relative to uninfected samples after 8 and 13 days post-infection, respectively [96]. Therefore, we addressed whether XMRV replication may alter tissue lymphocytes. Despite the apparent infection of the tissue, we did not observed changes in the percentage of the main lymphocyte populations after 9 days of infection (Figure 5). XMRV infection did not modify the percentage of CD3^+^ T-cells (63±11% and 64±8% in XMRV^−^ and XMRV^+^ tissue, respectively), nor CD4^+^ T-cells (47±7% vs 48±5%), nor CD8^+^ T-cells (20±12% vs 19±11%). The percentages of B cells were also similar between both tissues, CD19^+^ cells (29±16% vs 27±14%) (Figure 5). A deeper analysis of T-cell subsets showed that XMRV infection did not modify the naïve/memory cell ratio, in both T and B cells, nor immune activation markers, evaluated by the expression of CD38 and HLA-DR in both CD4^+^ and CD8^+^ T-cells (data no shown). None of these parameters was modified after 28 days in culture (data no shown).

### Release of different chemokines upon XMRV tissue infection

In human lymphoid tissue infected with other viruses such as HIV and HHV-6 a modulation in the chemokine secretion by viral replication has been documented [90], [97], [98]. To address this possibility, five chemokines from a designed Human Chemokine kit, some of them previously described to be altered in patients with CFS [99], [100] were measured by using a quantitative cytometric bead array on the supernatants collected during the course of 22 days of viral infection The total production of those chemokines did not significantly change by XMRV infection in the absence or presence of antiviral drugs (Figure 6).

### Recovery of replication-competent XMRV from supernatants of infected tonsils

The increase in DNA and viral RNA together with the presence of hypermutated sequences suggests an active replication. To evaluate if the supernatant of infected tonsils histocultures contained replication-competent virus, we performed a viral infectivity assay using DERSE XMRV indicator cells, which were infected with those supernatants. The indicator cell line DERSE XMRV will only produce GFP following infection with a replication-competent virus since the *gfp* gene is functionally reconstituted upon reverse transcription. DERSE cells were infected with 50 µL of supernatants harvested at day 16 and 22 (donor 402, Figure 3B) which contained 23,000 and 99,000 copies of XMRV RNA, respectively and with XMRV from 22Rv1 supernatant stock (40,000 copies of RNA) and the GFP expression was monitored by fluorescence microscopy every 2–3 days of infection. GFP^+^ cells were detected in the control culture (infected with 22Rv1 supernatant stock) as early as 3 days (data not shown) and a high number of cells were positive at day 7 post-infection (Figure 7A). At this time, GFP expression was also observed when cells were infected with the supernatants of infected tonsil, (Figure 7A middle and lower panels). No GFP expression was detected in mock-infected cells. To quantify the percentage of cells infected with each supernatant, DERSE cells were harvested and analyzed by flow cytometry at different days after infection. At 7 days post-infection a high number of GFP^+^ cells (28%) were detected when a 22Rv1 supernatant stock was used (Figure 7B). In the infections with the supernatants from day 16 and 22, and in agreement with the data observed by microscopy, a lower number of GFP^+^ cells were detected (4% and 7%, respectively), although the levels were significantly higher than those observed in uninfected wells (Figure 7B) at day 7. Following culture passage, an increase in the number of GFP+ cells was observed in all the infections, reaching similar values in the infections with both supernatant than in the infection with the 22Rv1 stock (21% and 27% of GFP+ cells with supernatants from day 16 and 22, respectively, and 29% of GFP+ cells with 22Rv1 supernatant. Figure 7B). The results from this viral infectivity assay suggested that XMRV viral particles, which were able to replicate and spread were presents in the tonsillar supernatants.

Furthermore, we analyze the ability of tonsillar supernatants to re-infect a new histoculture. In order to maximize the infection, tissue from two different donors was infected at day 0 and re-infected with the same amount of virus at day 1. We use each time 45 µL (5 µL for each tissue block) of XMRV 22Rv1 stock (4×10^8^ copies/mL), donor 402 supernatant day 16 (4.7×10^5^ copies/mL) and donor 402 supernatant day 22 (2×10^6^ copies/mL) (see Figure 3A). After 28 days of infection we observed a very large number of copies of proviral DNA in tissue cells infected with 22Rv1 supernatant and almost a total inhibition with AZT. However, none of the tonsil supernatants used to re-infect the tissue was able to infect this new tissue and only a residual amount of proviral DNA (even lower than that detected in the infection of 22Rv1+AZT) was observed, mainly in the supernatant from day 22 (Figure 8A). Consistent with the lack of detection of proviral DNA in tissue cells, we found only a residual level of both, proviral DNA in cells migrating out of the tissue (Figure 8B) and XMRV RNA released into the medium (Figure 8C).

## Discussion

During the last year an enormous amount of data about the origin and the prevalence of XMRV have indicated that this virus has a recombinant origin and it is not circulating in the human population [11]–[17], [23]–[33], [36]–[47], although many questions about the biology and physiopathology of this virus remain still unclear.

Despite all these data and considering, i) the susceptibility of humans cells to the XMRV infection [53], [101], [102], ii) the contradictory data on experimental infection of macaques by XMRV [86], [87], iii) the high-titer production of XMRV by the 22Rv1 cell line, widely used in laboratory, and iv) the existence of XMRV/human contacts in laboratory personnel involved in cell culture facilities, it could be relevant to develop new experimental models for the study of XMRV pathogenesis in humans alternative to the use of non-human primates.

The main objective of the present study was to investigate whether the human lymphoid tissue might be a target for XMRV derived from 22Rv1 supernatant. We used histocultures of human tonsillar tissues that have been reported as a useful model for studying various aspects of the pathogenesis of other viruses [88], [90], [103]. The *ex vivo* infection with XMRV allowed us to assess the replication of the virus, in addition to the pathogenic effects of this infection in human lymphoid tissue.

Tonsillar tissue after 14–22 days of infection become efficiently and specifically infected by XMRV. In contrast to previously described findings using *in vitro* infected PBMCs, the proviral copy numbers in our *ex vivo* infected histoculture increased overtime from day 2 to day 22 which could indicate that new target cells become infected throughout the culture. This increase in the proviral DNA content was concomitant to the increase in XMRV RNA released into the culture medium, suggesting that this XMRV RNA is associated with infectious particles that may lead to the spread of the infection. When the presence of infectious viral particles in the supernatants of infected tonsils was evaluated, we observed that we could recover replication-competent XMRV from these supernatants by infecting the indicator DERSE XMRV cell line. In contrast, those supernatants were unable to establish an infection in fresh lymphoid tissue, suggesting that with the passage of the virus through several rounds of infection, the innate antiviral restriction factors may abrogate its infectivity. Nevertheless, it should be noted that these supernatants have a much lower amount (2 to 3 log-copies of RNA/mL less) that the 22Rv1 supernatant used as a positive control of infection. Indeed, a high viral titer is required to establish an infection in human PBMCs [17], [54], so it is possible that viruses released by tonsils, although infectious in the DERSE assay, did not reach a sufficient titer to establish a new histoculture infection. In addition, the increase in proviral copy numbers, both in the tissue and the migrating cells, could be fully explained by cell divisions of infected cells, which in turn would lead to an increase in the level of released XMRV RNA.

Although the absolute levels of XMRV RNA released varied from donor to donor, the replication was similar to those observed in tonsillar tissue infected with some strains of HIV (R5 strains) (ranging between 2 and 5×10^6^ RNA copies/mL) [97], but lower than viral production of more pathogenic strains of HIV (×4 strains), which range from 15 to 60×10^6^ RNA copies/mL [97]. However, for HIV has never been reported whether this amount of virus produced is sufficient to establish a new productive infection in a new histoculture.

Our observation that we could not establish a new infection in tonsillar tissue may indicate that, in *ex vivo* infected human lymphoid tissue, the virus is restricted by innate host restriction factors such the APOBEC family, since hypermutation, the introduction of excessive G-to-A substitutions by those host proteins that impair the viral replication, has also been observed in our experimental model. The sequence analysis of our clones showed that 19% of the sequences were hypermutated. This percentage, although lower than the 77 and 65% of hypermutated sequences found with the infection of two APOBEC 3G/3F positive cell lines (CEM and H9, respectively) [83], is similar with the level of hypermutagenesis described in the infection of PHA-stimulated PBMCs [54]. In addition, the mutation frequencies obtained from our sequences (3×10^−3^/nt) were higher than those found in 22Rv1 proviral DNA (4.9×10^−4^/nt) or with the infection of an APOBEC 3G/3F negative cell (CEM-SS) (8.4×10^−4^/nt), although they were similar to those found in the infection of H9 cells (6.6×10^−3^/nt) [83]. Moreover, although G-to-A mutations can occur in one of four different dinucleotide contexts (GG, GA, GC or GT), APOBEC 3G induces twice as many GG-to-AG as GA-to-AA changes [81], while APOBEC 3F primarily induce GA-to-AA [76]. In tissue, most of the G-to-A changes occurred in a GG dinucleotide context. Overall, our data indicate that XMRV infection would be restricted, mainly by APOBEC 3G, as described in *in vitro* infected PBMCs or human cell lines. Nonetheless, in lymphoid tissue, the virus could somehow be able to evade or overcome these restriction factors, as a low amount of infectious virus is released. There are various non-excluding explanations for this observation. The most obvious conjecture could be that the level of APOBEC 3G is lower in lymphoid tissue than in peripheral PBMCs and not enough to block virus replication. However, all APOBEC family members are expressed widely in hematopoietic cell populations and in tissues, particularly in tonsils [95]. Secondly, despite the fact that our data show that mechanically isolated lymphocytes from tissue and migrating cells are XMRV DNA^+^, the cell population responsible for the viral production remains unknown. In our histocultures, XMRV could infect other cell types that do not express these restriction factors, being a non-lymphoid cell the major viral target as it was shown in prostate cancer tissues [3] or in tissues from experimentally infected monkeys [86]. Further work is necessary to define the cellular population that would sustain this low viral production in *ex vivo* infected tonsils.

In *ex vivo* HIV infected tonsils, virus replication causes a profound depletion of the viral target cells (CD4^+^ T cells). In our histocultures, we did not observe changes in the percentages in any of the major lymphocyte populations or modifications in the release of inflammatory chemokines. Yet, in XMRV infected macaques the virus did not induce any lymphocyte depletion, rather there was an increase in the frequency of circulating B and NK cells in blood [86].

In conclusion, in the absence of any confirmed human XMRV infection and with the conflicting results of infection in animal models, which may not accurately mimic human XMRV infection, in our *ex vivo* cultured human lymphoid tissue, XMRV is able to infect tissue cells and produce infectious viruses, even though they were unable to establish a new infection in fresh tonsillar tissue. The XMRV replication is largely controlled by innate antiviral restriction factors and probably further contained by the adaptative immune response, although the long term presence of proviral DNA and viral RNA in the lymphoid tissue remains absolutely unknown. Hereby, laboratories working with XMRV producing cell lines should be aware of the potential biohazard risk of working with this replication-competent retrovirus.
